# Sleep Disturbances in Children With Atopic Dermatitis: A Scoping Review

**DOI:** 10.1177/12034754231159337

**Published:** 2023-03-07

**Authors:** Dong Goo Lee, Xi Yao Gui, Ilya Mukovozov, Patrick Fleming, Charles Lynde

**Affiliations:** 1 12358 Faculty of Medicine, University of British Columbia, Vancouver, British Columbia, Canada; 2 12358 Department of Dermatology and Skin Science, University of British Columbia, Vancouver, British Columbia, Canada; 3 12366 Division of Dermatology, University of Toronto, Toronto, ON, Canada; 4 The Lynde Institute for Dermatology, Markham, ON, Canada

**Keywords:** atopic dermatitis, eczema, sleep, adolescent, teen, child

## Abstract

Atopic dermatitis (AD) is associated with various quality of life concerns including poor sleep. Sleep impairments in children with AD are associated with increased risk of short stature, metabolic syndrome, mental illness and neurocognitive dysfunction. Although the association between AD and sleep disturbance is well established, the specific types of sleep disturbance in pediatric AD patients and their underlying mechanisms are not fully understood. A scoping literature review was performed to characterize and summarize the types of sleep disturbance in children (less than 18 years of age) with AD. 31 papers met inclusion criteria and extracted data were analyzed in an iterative manner. Two types of sleep disturbances were found to be more prevalent in pediatric AD patients in comparison to controls. One category was related to loss of sleep (increased frequency or duration of awakenings, increased sleep fragmentation, delayed sleep onset, decreased total sleep duration, and decreased sleep efficiency). Another category was associated with unusual behaviors during sleep (restlessness/limb movement/scratching, sleep-disordered breathing including obstructive sleep apnea and snoring, nightmares, nocturnal enuresis and nocturnal hyperhidrosis). Some mechanisms underlying these sleep disturbances include pruritus and induced scratching and increased proinflammatory markers induced by sleep loss. Sleep disturbance appears to be associated with AD. We recommend clinicians to consider interventions that may reduce sleep disturbances in children with AD. Further investigation of these sleep disturbances is needed to elucidate pathophysiology, develop additional treatments, and reduce negative impacts on the health outcomes and quality of life in pediatric AD patients.

## Introduction

Atopic dermatitis (AD) is a chronic, relapsing, and pruritic skin condition that begins in the first 5 years of life in 90% of patients.^1^ AD is common, affecting 10 to 20% of children in developed countries.^1^ AD usually presents in a characteristic age-dependent distribution with facial, scalp, and extensor involvement in infants and young children, and predominant flexural involvement in older children and adults.^1^

Children with AD and their parents report sleep disruption, most often caused and exacerbated by pruritus, to be one of the most problematic aspects of the condition.^2^ In previous studies, sleep disruption has been found to be positively correlated with AD severity.^2^ Common features of disrupted sleep in AD patients include daytime sleepiness, difficulty falling asleep, nighttime awakenings, greater number of shifts in sleep stages, less non-rapid eye movement sleep, difficulty waking in the morning, and tiredness and irritability during the day.^3-5^ Despite the importance of sleep in patients with AD, sleep is often viewed as a secondary focus of disease control in studies assessing therapeutics in patients with AD, and few studies have evaluated treatment methods specifically aimed at improving sleep.^5^ Presently, there is no consensus or available guidelines on how best to manage sleep problems in these patients.^5^

The pathophysiology of sleep disturbance in children with AD is poorly understood and likely involves intertwined relationships between pruritus and scratching as well as the circadian rhythm, immune system, and environment.^5^ Sleep disturbances are common among children with AD, reported in 47% to 80% of patients and is a major factor leading to an impaired quality of life.^6^ The negative consequences of sleep disturbance in children with AD include impaired neurocognitive function, higher rates of behavioral problems, changes in mood, ADHD, emotional and conduct problems, and short stature.^5^ Therefore, further studies are needed for a better understanding of the types of sleep disturbances in pediatric AD patients and their underlying pathophysiology in order to formulate better treatment strategies accordingly.^5^ Our primary objective is to provide a precursory evaluation regarding the range of existing literature regarding the sleep disturbances in pediatric patients with AD.

## Methods

A scoping review was conducted to identify and synthesize relevant research. This scoping review was produced in adherence to the Preferred Reporting Items for Systematic Reviews and Meta-Analyses (PRISMA) extension for scoping reviews (Supplemental Figure 1).

### Search Strategy, Eligibility Criteria and Study Selection

A broad and comprehensive combination of search terms for sleep, sleep disorders, AD and children were incorporated into the search strategy, which is outlined in Supplemental Tables 1 and 2. This search strategy was implemented using the Ovid interface on Medline (last accessed Jan 1, 2022) as well as PsycINFO (last accessed October 12, 2022). An included study necessarily contained data involving children, defined as individuals under the age of 18, with a clinical diagnosis of atopic dermatitis as defined by the manuscript. Studies were included without limitation regarding their type of intervention or control group. Additionally, studies with any outcome related to sleep disturbances were included except studies only containing data measuring sleep disturbance in a general sense without further characterization. For example, patient data from the SCORAD (SCORing Atopic Dermatitis) scale were excluded as this scale only has a general item pertaining to sleep disturbance which requests patients to rate sleeplessness on a scale of 0 (“none”) to 10 (“worst possible”). It was required that the full manuscript was available in English, and the study type was qualitative or quantitative, patient survey, observational study, case-control study or randomized control trial. Studies were excluded if they involved less than 10 patients (e.g., case reports or case series with 9 or less patients), surveys completed by health care providers without patient reported data or outcomes, protocol papers, or non-human studies.

Title, abstract and full-text screening was conducted independently in duplicate by two reviewers (DGL and XYG) using Covidence online systematic review software (www.covidence.org). Disagreements were resolved by consensus. The references of included studies were reviewed to ensure relevant studies were not missed.

Two reviewers (DGL and XYG) independently extracted data from included studies using a standardized extraction form with categories including title, authors, year of publication, study design, main findings, and reasons for exclusion. Reviewers cooperated to synthesize and organize the results of extraction.

## Results

### Data Extraction and Synthesis

The search led to 568 total results, and 31 papers were included in the final review (Supplemental Figure 1). A summary of findings is provided in Supplemental Table 3. Studies were only included if they contained data pertaining to specific types of sleep disturbances in children diagnosed with AD.

Review of the results revealed two clusters of sleep disturbances which were found to have an increased prevalence in pediatric patients with AD in comparison to control populations. One category of sleep disturbances revolved around loss of sleep, which consisted of higher frequency and prolonged duration of awakenings, increased sleep fragmentation, delayed sleep onset, shortened total sleep duration, and decreased sleep efficiency. In contrast, the second cluster of sleep disturbances involved unusual behaviors demonstrated during sleep, consisting of increased movement (including restlessness, limb movement or scratching), sleep disordered breathing (including obstructive sleep apnea or snoring), nightmares, nocturnal enuresis, and nocturnal hyperhidrosis. This categorization resembles the grouping of insomnias and parasomnias utilized by sleep disorders literature and aims to facilitate the readers’ conceptualization of the sleep disturbances discussed in this paper without implying formal diagnoses.

The results involve studies with varying methods and terminologies for investigating sleep disturbances. In actigraphy, subjects wear specialized wristwatches to record bodily movement patterns to assess whether an individual is likely asleep. Polysomnography (PSG) is the recording of various physiological signals of various organs including the brain, eyes, and muscles which allows for the measurement of their activity levels and objective assessment of an individual’s sleep. Additionally, sleep fragmentation refers to arousals, which are abrupt shifts in electroencephalographic (EEG) frequency suggestive of an awake state, occurring after 10 consecutive seconds of sleep and lasting longer than 3 seconds. This sleep modality may be measured by electroencephalography, or by actigraphy in which case movements are used as a proxy for wakefulness and the degree of sleep fragmentation is expressed as a sleep fragmentation index (SFI).

Furthermore, the results elucidate some of the mechanisms underlying these sleep disturbances, such as pruritus and induced scratching, sleep anxiety and increased proinflammatory markers induced by sleep loss.

### Cluster 1: Sleep Disturbances in Pediatric AD Patients Involving Loss of Sleep

#### Higher frequency and prolonged duration of awakenings, and increased sleep fragmentation

There is evidence linking AD to impaired sleep in children, particularly related to an increased number of awakenings during sleep as well as a prolonged duration of wakefulness prior to being able to return to sleep.^7-13^ Parental surveys of children revealed that more pediatric AD patients woke up over 3 times in a night compared to controls (52.2%, 40%, *P* = .4) and stayed awake longer than 1 hr after initial sleep onset (41.3%, 11.7%, *P* = .005).^7^ Additionally, there is an apparent association between the degree of these sleep disturbances and the disease severity of AD.^9^ Kahn et al. (2020) demonstrated that parents of children with AD responded that their child on average had 3.6 ± 4.4 awakenings per night based on questionnaires, but this was significantly less than the average number of awakenings measured by actigraphy (*P* = .008).^8^ Supporting these findings, a significant correlation has been found between a pediatric AD patient’s SCORAD score, which evaluates the severity of AD, and the parentally reported number of awakenings of the patients.^13^ Fishbein et al. (2018) reported that pediatric AD patients’ total duration of awakening after initial sleep onset, measured by actigraphy, was greater than that of controls (103 minutes vs. 50 minutes, *P* < .01).^14^ Furthermore, Reuveni et al. (1999) and Chang et al. (2014) discovered significantly higher frequency of arousals, and by extension more extensive sleep fragmentation, in AD patients compared to controls using EEG and actigraphy (24.1, 6.2 arousal events per hour, *P* < .001), and SFI (22.1, 17.0, *P* = .004), respectively.^11,12^ Overall, findings indicate increased sleep fragmentation as well as awakenings occur more often and for longer periods for children with AD compared to controls.

#### Delayed sleep onset

Multiple studies demonstrated delayed sleep onset or sleep latency in children with AD, which refers to a prolonged duration of time taken for an individual to transition from wakefulness to somnolence. Danish parental questionnaires revealed that 65.1% of children with severe AD reported difficulties falling asleep every day or several times a week compared to 7.0% of children with clear/almost clear AD (*P* < .0005) while a parental questionnaire study in Saudi Arabian children found that those with mild or moderate AD had significantly increased sleep latency compared to those with severe AD (*P* < .005).^15,16^ This is supported by Dahl et al. (1995) who demonstrated that parents of children with AD compared to controls showed lower ratings in response to the Likert scale item, “Does your child fall asleep easily?” (*P* = .001).^9^ Additionally, Dogan et al. (2017) demonstrated an increased sleep latency in pediatric AD patients compared to controls using a parental questionnaire (28.7, 25.2 minutes, *P* = .2), although this difference was not significant.^7^ Finally, the previously mentioned study by Chang et al. (2014) involving actigraphy and polysomnography of pediatric AD patients and controls showed a significantly greater mean sleep latency in pediatric AD patients (45, 27 minutes, *P* < .001).^12^ Overall, children with AD have increased duration and frequency of sleep latency compared to controls demonstrated by both qualitative and quantitative measures.

#### Decreased total sleep duration

Total sleep duration has been found to be significantly decreased in AD children compared to non-AD children. Chen et al. (2021) performed a cross-sectional survey in China with the adolescent sleep disturbance questionnaire and found that an increased risk of eczema was correlated with a sleep duration of less than 8 hr on weekends (*P* = .04).^17^ Furthermore, Emerson et al. (2000) conducted parental questionnaires of children aged 1-5 years and demonstrated that 4.5% had experienced significant sleep duration loss due to pruritus and scratching from AD.^18^ Anuntaseree et al. (2012) conducted a parental cross-sectional survey to find that sleep duration was reduced in infants with severe AD compared to controls (542 ± 67 versus 569 ± 62 minutes, *P* = .02).^19^ In another study by Shani-Adir et al. (2009), sleep quality in 94 children aged 4-10 years was evaluated using the parent-reported, 33-item Children’s Sleep Habits Questionnaire^20^. As a result, the AD group showed significantly worse sleep quality than controls in the following areas: sleep duration, bedtime resistance, parasomnias, and daytime sleepiness (all *P* ≤ .05).^20^ Finally, a study by Chen et al. (2022) that sampled Chinese children from ages 7-12 showed that children without eczema slept an average of 9.53 hr per night, while children with eczema slept an average of 9.42 hr per night (*P* < .001).^21^

#### Decreased sleep efficiency

Sleep efficiency is commonly defined in the literature as the ratio of total sleep time to the total time spent in bed, expressed as a percentage.^22^ An individual’s sleep efficiency may be decreased by either decreasing the total sleep time or increasing the total time spent in bed awake. The significance of sleep efficiency is prominent in insomnia research, as it captures the issue of spending an increased proportion of time in bed trying to fall asleep, both initially and after awakenings.^23^ Several studies report decreased sleep efficiency in children with AD. Kahn et al. (2020) showed that for pediatric AD patients, mean nocturnal sleep efficiency was 86 ± 6%, being reduced in 50% of patients.^8^ Additionally, Stores et al (1998) compared home polysomnography results for 20 children with AD and controls and discovered that the sleep efficiency was reduced in children with AD (92.8%, 98.3%, *P* < .001).^24^ Overall, these results suggest that sleep efficiency is lower in children with AD, especially in those with a severe disease state.

### Cluster 2: Sleep Disturbances in Pediatric AD Patients Involving Unusual Behaviors

#### Increased movement including restlessness, limb movement, or scratching

Pediatric patients with AD reportedly demonstrate higher frequency of unusual behaviors including movement. Fishbein et al. (2018) analyzed parental responses to the Pediatric Sleep Questionnaire and demonstrated that children with AD noted significantly higher frequencies of restless sleep compared to controls (*P* < .01).^14^ Reuveni et al. (1999) used direct observation, video monitoring, and scratch electrodes to show that AD patients experienced bouts of scratching that ranged from 1 to 19 times per night (1.8 ± .6 bouts per hour).^11^ Interestingly, only 15% of overall arousals and awakenings were associated with PSG events such as scratching or other movements, or apnea.^11^ Benjamin et al. (2004) used limb-worn digital accelerometers and infrared video recordings to produce the finding that scratching or restlessness was 2 to 3 times more frequent in children with AD than control subjects (*P* < .01).^25^ Finally, Chang et al. (2014) confirmed these findings with a study using actigraphy and polysomnography, revealing that there was significantly more movement during sleep in the patients with AD compared with the controls, specifically in terms of the percentage of mobility during sleep time (*P* < .05), as well as limb movement index (*P* < .001).^12^ In contrast, Dahl et al. (1995) investigated children’s sleep habits and behaviors of using the Child Sleep Behavior Scale and no significant differences were found for other various types of unusual movement including bruxism, head banging, body rocking, or sleepwalking (*P* > .05).^26^ Overall, while findings are mixed, there exists recent qualitative and objective data supporting the increased frequency of scratching and limb movement in children with AD compared to controls.

#### Obstructive sleep apnea and snoring

Obstructive sleep apnea (OSA) is a disorder involving collapses of the upper airway during sleep, which is the main pathophysiologic mechanism of chronic sleep fragmentation and intermittent hypoxia.^27^ Although habitual snoring is often associated with OSA, it alone is also strongly associated with having underlying atopy.^28,29^ Chng et al. (2004) demonstrated that the odds ratio of a child with asthma, allergic rhinitis, and/or AD to having habitual snoring was 7.45 (3.48, 15.97), and in particular, the odds ratio of a child with AD to habitual snoring was 1.80 (1.28, 2.54).^29^ Hu et al. (2018) evaluated the association between OSA and AD in children in a population-based cohort for a follow up period of approximately 5 years.^27^ After adjusting for age, sex, urbanization level, and comorbidities, patients with AD had a higher risk of OSA than controls, with an adjusted HR of 1.86 (1.43, 2.42).^27^ Together, these findings support that there is a significant correlation between AD and OSA, and that individuals with AD are at increased risk for snoring and developing OSA. Neurocognitive dysfunctions associated with OSA may result from frequent arousals and fragmented sleep, such as impaired memory and concentration and irritable mood.^30^

#### Nightmares

Nightmares are defined as dreams with strong negative emotions which awaken the dreamer.^31^ Ramirez et al. (2019) conducted a population-based cohort study and concluded that for children between ages 2-10, 26.7%-49.5% of children experienced regular nightmares.^32^ In the survey findings of Dahl et al. (1995), children with AD aged 6-11.5 years did not significantly differ from controls regarding the frequency of nightmares, sleep terrors, or frightening dreams (*P* > .05).^26^ Additional studies are needed to investigate the relationship between parasomnias and AD. Currently, there is mixed evidence regarding the increased prevalence of nightmares in children with AD. The impact is that children with chronic nightmares have been shown to have more emotional symptoms, hyperactivity, conduct problems, and peer problems than children without or with transient nightmares.^31^

#### Nocturnal hyperhidrosis

Hyperhidrosis is an excessive generalized or localized production of sweat.^33^ A parental survey study by Camfferman et al. (2010) involving Sleep Disturbance Scale for Children found that a higher percentage of children with eczema compared to controls were above the clinical cut-off criteria (T-score >70) for sleep hyperhidrosis (9% [7/77] vs. 7% [2/30]).^34^ Furthermore, sweating can in turn further exacerbate itching in patients with AD as sweat is a major trigger of itch in AD.^34^ A study by Ono et al. (2018) compared the properties of sweat between AD and healthy subjects who were induced to sweat in a sauna.^35^ In sweat from subjects with AD, the concentration of LL-37, an antimicrobial peptide, varied greatly among individuals.^35^ As LL-37 is cytotoxic, it can be imagined that sweat with high sweat concentrations of LL-37 may promote inflammation and cause itching in subjects with AD.^35^

#### Nocturnal enuresis

Childhood nocturnal enuresis (NE) refers to the symptoms of intermittent urinary incontinence during sleep.^36^ NE leads to nights of disturbed sleep that causes mood disturbance and daytime sleepiness.^37^ Tsai et al. (2017) found that children with atopic dermatitis had a significantly higher odds ratio of 1.23 (1.05, 1.43, *P* = .008) of having NE compared to controls after adjusting for confounding variables.^38^ However, the precise pathophysiology linking AD and NE is unknown.^38^ One proposed mechanism is that excessive parasympathetic nervous system activation after cycles of allergic responses targets the bladder, leading to detrusor muscle instability and overactive bladder.^38^ In support of this hypothesis, recent studies have found that the degree of parasympathetic nervous system overactivity was correlated with disease severity in children with allergy and NE.^38^

## Discussion

Numerous studies have hypothesized or demonstrated the association of sleep disturbance in children with AD with negative outcomes, including impaired cognitive functioning (including verbal comprehension, perceptual reasoning, working memory and concentration), increased frequency of absence from daycare of school due to AD, daytime sleepiness, behavioral problems (such as excessive dependency, clinginess and fearfulness), psychiatric comorbidities such as (anxiety, stress, labile mood, depression and ADHD), headache, impaired child daytime functioning (such as impaired school performance and impaired social activities), as well as growth impairment and obesity.^15,26,39-45^

Various techniques have been employed to investigate the types of severity of sleep impairment in AD, including questionnaires completed by patients or their parents, videography, actigraphy, electroencephalography and polysomnography.^7,10,11^ In comparison to previous studies which have postulated the pathophysiology behind certain types of sleep disorders associated to AD, this study is a preliminary evaluation of the extent of available research regarding the types of sleep disturbances in pediatric AD. Our findings reveal that children with AD may suffer from sleep-loss related disturbances (higher frequency and prolonged duration of awakenings, increased sleep fragmentation, delayed sleep onset, shortened total sleep duration, and decreased sleep efficiency) as well as disturbances related to unusual behavior (increased movement including restlessness, limb movement or scratching, and sleep disordered breathing including obstructive sleep apnea or snoring, as well as nightmares, nocturnal enuresis, and nocturnal hyperhidrosis).

The findings of our study suggest that there are factors beyond itch and scratching which contribute to the diverse types of sleep disturbances experienced by pediatric AD patients. In fact, Reuveni et al. (1999) provided data from direct observation, video monitoring, scratch electrodes, EEG and PSG showing that only 15% of overall arousals and awakenings were associated with PSG movements like apneas, or jerks and movements such as scratching.^11^ Further research is required to elucidate the pathophysiology of sleep disturbances in AD, as current research does not extend significantly beyond pruritus and scratching as the main explanations.^4,17^

The main results of our investigation of the types of sleep disturbances in children with AD are summarized in Supplemental Table 3. It is primarily notable that pediatric AD patients would benefit from receiving treatment for their condition as AD severity has been linked to severity of sleep disturbance and improvement in AD severity as been shown to reduce sleep disturbances.^8,38,46-48^ Additionally, general measures to facilitate sleep should be adhered to, such as sleep hygiene practices. Reducing the severity of pediatric AD and its associated sleep disturbances may additionally reduce the impact of the numerous aforementioned postulated and established negative outcomes.

Due to the scarcity of literature investigating the pathophysiology of sleep disturbances in children with AD, this study is limited to a general overview of the types of such sleep disturbances. A limitation of our findings is that they are partially based on proxy measures such as questionnaires completed by parents instead of patients, as well as actigraphy which cannot determine whether an individual is certainly asleep but is only able to draw likely conclusions based on an individual’s pattern of movement. Furthermore, some discordances in the evaluation of sleep disturbance have been found in studies that have recorded both subjective and objective measures. A study by Kahn et al. (2020) demonstrated that parents underestimated both sleep duration and number of awakenings in their children (mean age of 5.6 ± 5.3 years) on questionnaires compared to measurements by actigraphy.^49^ This suggests that parental questionnaires may not most accurately reflect these parameters in children with AD^49^. Possible causes include parental tiredness, underrecognizing of child’s sleep characteristics and difficulties with monitoring children who sleep in separate rooms.^49^ Additional limitations include the study design of scoping reviews, the lack of study quality evaluation and furthermore the potential selection bias pertaining to included papers in this study. Despite the limitations of a scoping review, it allowed greater flexibility in exploring a broad topic including subthemes, such as nightmares and nocturnal hyperhidrosis in pediatric AD, which currently are lacking in sufficient quantity of data and may not currently appear to warrant additional analyses such as a risk of bias evaluation.

## Conclusion

This study summarizes numerous types of sleep disturbances in children with AD which are associated to multiple negative health outcomes. Future research pertaining to the pathophysiology and management of these sleep disturbances is needed to improve the health and quality of life of children with AD.

## Supplemental Material

Table S1 - Supplemental material for Sleep Disturbances in Children With Atopic Dermatitis: A Scoping Review Click here for additional data file. Supplemental material, Table S1, for Sleep Disturbances in Children With Atopic Dermatitis: A Scoping Review by Dong Goo Lee, Xi Yao Gui, Ilya Mukovozov, Patrick Fleming and Charles Lynde in Journal of Cutaneous Medicine and Surgery
