# Supplementary material for: Sleep Disturbances in Children With Atopic Dermatitis: A Scoping Review
Source: J Cutan Med Surg. 2023 Mar 7;27(2):157–64. doi: 10.1177/12034754231159337 (PMC10068401; doi:10.1177/12034754231159337)
Supplement: Table S1 - Supplemental material for Sleep Disturbances in Children With Atopic Dermatitis: A Scoping Review [file sj-docx-1-cms-10.1177_12034754231159337.docx]

**Sleep Disturbances in Children with Atopic Dermatitis: A Scoping Review**

**Supplementary Material**

**Identification of studies via databases and registers**

Records removed *before screening*:

Duplicate records removed (n = 24)

Records marked as ineligible by automation tools (n = 0)

Records removed for other reasons (n = 0)

Records identified from:

Databases (n = 568)

Registers (n = 0)

**Identification**

Records screened

(n = 544)

Records excluded

(n = 418)

Reports sought for retrieval

(n = 126)

Reports not retrieved

(n = 0)

**Screening**

Reports assessed for eligibility

(n = 126)

Reports excluded:

Irrelevant outcomes (n = 69)

Foreign language (n = 3)

Full text not available (n = 3)

Duplicates (n = 2)

Meets exclusion criteria (n = 18)

Studies included in review

(n = 31)

Reports of included studies

(n = 31)

**Included**

Supplementary Figure 1. PRISMA Flow Diagram

Supplementary Table 1. MEDLINE Search Strategy

TX ((Sleep*) OR (REM Sleep Behavior Disorder) OR (Sleep Aids) OR (Sleep Apnea) OR (Sleep Bruxism) OR (Sleep Deprivation) OR (Sleep Disorders) OR (Sleep Hygiene) OR (Sleep Initiation and Maintenance Disorders) OR (Sleep Latency) OR (Sleep Medicine) OR (Sleep Stages) OR (Delta Sleep-Inducing Peptide) OR (Sleep Wake Disorders) OR (REM Sleep Parasomnias) OR (Sleep Arousal Disorders) OR (Sleep Paralysis) OR (Sleep-Wake Transition Disorders) OR (Sleep Phase Chronotherapy)) AND ((Atopic dermatiti*) OR (Eczema*) OR (Neurodermatiti*) OR (dermatitis atopi*) OR (besnier's prurigo) OR (besniers prurigo) OR (infantile eczema) OR (childhood eczema)) AND ((Child*) OR (Adolescen*) OR (Juvenil*) OR (teen*))

Supplementary Table 2. PsycINFO Search Strategy

| 1. | sleep*.mp. |
| --- | --- |
| 2. | exp Sleep/ |
| 3. | exp REM Sleep Behavior Disorder/ |
| 4. | exp Sleep Aids, Pharmaceutical/ |
| 5. | exp Sleep Apnea, Central/ |
| 6. | exp Sleep Apnea, Obstructive/ |
| 7. | exp Sleep Apnea Syndromes/ |
| 8. | exp Sleep Bruxism/ |
| 9. | exp Sleep Deprivation/ |
| 10. | exp Sleep Disorders, Circadian Rhythm/ |
| 11. | exp Sleep Disorders, Intrinsic/ |
| 12. | exp Sleep Hygiene/ |
| 13. | exp "Sleep Initiation and Maintenance Disorders"/ |
| 14. | exp Sleep Latency/ |
| 15. | exp Sleep Medicine Specialty/ |
| 16. | exp Sleep, REM/ |
| 17. | exp Sleep, Slow-Wave/ |
| 18. | exp Sleep Stages/ |
| 19. | exp Delta Sleep-Inducing Peptide/ |
| 20. | exp Sleep Wake Disorders/ |
| 21. | exp REM Sleep Parasomnias/ |
| 22. | exp Sleep Arousal Disorders/ |
| 23. | exp Sleep Paralysis/ |
| 24. | exp Sleep-Wake Transition Disorders/ |
| 25. | exp Sleep Phase Chronotherapy/ |
| 26. | exp Dermatitis, Atopic/ or atopic dermatiti*.mp. |
| 27. | exp Eczema/ or eczema*.mp. |
| 28. | exp Eczema, Dyshidrotic/ |
| 29. | exp Neurodermatitis/ or neurodermatiti*.mp. |
| 30. | exp Dermatitis, Atopic/ or dermatitis atopi*.mp. |
| 31. | besnier's prurigo.mp. |
| 32. | besniers prurigo.mp. |
| 33. | infantile eczema*.mp. |
| 34. | childhood eczema*.mp. |
| 35. | exp Child/ or child*.mp. |
| 36. | exp Adolescent/ or adolescen*.mp. |
| 37. | juvenil*.mp. |
| 38. | teen*.mp. |
| 39. | 1 or 2 or 3 or 4 or 5 or 6 or 7 or 8 or 9 or 10 or 11 or 12 or 13 or 14 or 15 or 16 or 17 or 18 or 19 or 20 or 21 or 22 or 23 or 24 or 25 |
| 40. | 26 or 27 or 28 or 29 or 30 or 31 or 32 or 33 or 34 |
| 41. | 35 or 36 or 37 or 38 |
| 42. | 39 and 40 and 41 |

Supplementary Table 3. Summary of Results

| **Author (Year)**  **Study title** | **Design** | **Number of subjects** | **Control population** | **Assessment modalities** | **Relevant findings** |
| --- | --- | --- | --- | --- | --- |
| *Higher frequency and prolonged duration of awakenings, and increased sleep fragmentation* | | | | | |
| Dogan (2017)  Sleep patterns of young children with newly diagnosed atopic dermatitis. | Case-control | 46 cases  60 controls | No AD | Brief Infant Sleep Questionnaire (BISQ) | More pediatric AD patients woke up over 3 times in a night compared to controls (52.2% vs 40%, p=0.4) and stayed awake longer than one hour after initial sleep onset (41.3% vs. 11.7%, p=0.005). |
| Kahn (2020)  Sleep quality in children with atopic dermatitis during flares and after treatment. | Prospective case-crossover | 10 cases | Not applicable | Brief Infant Sleep Questionnaire (BISQ)  Actigraphy | Parents of children with AD responded that their child on average had 3.6 ± 4.4 awakenings per night based on questionnaires, but this was significantly less than the average number of awakenings measured by actigraphy (p=0.008). |
| Fishbein (2018)  Sleep disturbance in children with moderate/severe atopic dermatitis: A case-control study. | Case-control | 19 cases  19 controls | No AD | Actigraphy  Pediatric Sleep Questionnaire | Pediatric AD patients’ total duration of awakening after initial sleep onset, measured by actigraphy, was greater than that of controls (103 minutes vs. 50 minutes, p<0.01) |
| Reuveni (1999)  Sleep Fragmentation in Children with Atopic Dermatitis. | Case-control | 14 cases  9 controls | No AD | Polysomnography  Scratch electrodes  Self-reported questionnaires completed by caregivers | There were significantly higher frequencies of arousal in AD patients compared to controls (24.1 vs. 6.2 arousal events per hour, p<0.001). |
| Chang (2014)  Atopic Dermatitis, Melatonin, and Sleep Disturbance. | Case-control | 72 cases  32 controls | No AD | Actigraphy  Polysomnography  SCORAD | There were significantly higher frequencies of arousal in AD patients compared to controls (22.1 vs. 17.0 arousal events per hour, p=0.004). |
| *Delayed sleep onset* | | | | | |
| Gerner (2021)  Disease severity and trigger factors in Danish children with atopic dermatitis: a nationwide study. | Cross-sectional | 1343 cases | Not applicable | Questionnaire completed by caregivers  Parent-Oriented Eczema Measurement (POEM) tool | 65.1% of children with severe AD reported difficulties falling asleep every day or several times a week compared to 7.0% of children with clear/almost clear AD (p<0.0005). |
| Alzolibani (2014)  Impact of atopic dermatitis on the quality of life of Saudi children. | Cross-sectional | 630 cases | Not applicable | Arabic version of the Infants’ Dermatitis Quality of Life (IDQoL) index  SCORAD | Parents of children under 4 years of age with severe AD provided significantly higher ratings regarding “time to get to sleep” compared to children with moderate AD (p=0.000) or compared to children with mild AD (p=0.000). |
| Dahl (1995)  Sleep disturbances in children with atopic dermatitis. | Cross-sectional | 59 cases | Normative data | Child Sleep Behavior Scale | Parents of children with AD compared to controls showed significantly lower ratings in response to the Likert scale item, “Does your child fall asleep easily?” (p=0.001). |
| Dogan (2017)  Sleep patterns of young children with newly diagnosed atopic dermatitis. | Case-control | 46 cases  60 controls | No AD | Brief Infant Sleep Questionnaire | There was increased sleep latency in pediatric AD patients compared to controls (28.7 vs. 25.2 minutes, p=0.2), although this difference was not significant. |
| Chang (2014)  Atopic Dermatitis, Melatonin, and Sleep Disturbance. | Case-control | 72 cases  32 controls | No AD | Actigraphy  Polysomnography  SCORAD | There was a significantly greater mean sleep latency in pediatric AD patients than controls (45 vs. 27 minutes, p<0.001). |
| *Decreased total sleep duration* | | | | | |
| Chen (2021)  Associations of sleep characteristics with atopic disease: a cross-sectional study among Chinese adolescents. | Cross-sectional | 661 cases | Not applicable | Chinese version of the Adolescent Sleep Disturbance Questionnaire and Adolescent Sleep Hygiene Scale | An increased risk of eczema was correlated with a sleep duration of less than 8 hours on weekends (p=0.04). |
| Emerson (2000)  The Nottingham Eczema Severity Score: preliminary refinement of the Rajka and Langeland grading. | Cross-sectional | 290 cases | Not applicable | Nottingham Eczema Severity Score | 4.5% of patients with AD had experienced significant sleep duration loss (6 or more nights of average sleep loss per week over 12 months) due to pruritus and scratching. |
| Anuntaseree (2012)  Sleep quality in infants with atopic dermatitis: a community-based, birth cohort study. | Case-control | 148 cases  3937 controls | No AD | Interviews and a 3-day sleep-wake record completed by caregivers | Sleep duration was reduced in infants with severe AD compared to controls (542±67 versus 569±62 minutes, p=0.02). |
| Shani-Adir (2009)  The Relationship Between Sensory Hypersensitivity and Sleep Quality of Children with Atopic Dermatitis. | Case-control | 57 cases  37 controls | No AD | SCORAD  Children’s Sleep Habits Questionnaire  Short Sensory Profile | Patients with AD showed significantly worse sleep quality than controls in the following areas: sleep duration, bedtime resistance, parasomnias, and daytime sleepiness (all p≤0.05) |
| Chen (2022)  Association of delayed chronotype with allergic diseases in primary school children. | Case-control | 805 cases  4641 controls | No AD | International Study of Asthma and Allergies in Childhood (ISAAC) questionnaire | Children ages 7-12 were sampled in Shanghai, China. Children with no eczema slept an average of 9.53 hours per night, while children with eczema slept an average of 9.42 hours per night (p<0.001). |
| *Decreased sleep efficiency* | | | | | |
| Kahn (2020)  Sleep quality in children with atopic dermatitis during flares and after treatment. | Prospective case-crossover | 10 cases | Not applicable | Brief Infant Sleep Questionnaire (BISQ)  Actigraphy | For pediatric AD patients, mean nocturnal sleep efficiency was 86 ± 6%, being reduced in 50% of patients. |
| Stores (1998)  Physiological sleep disturbance in children with atopic dermatitis: a case control study. | Case-control | 20 cases  20 controls | No AD | Polysomnography | Sleep efficiency was significantly reduced in children with AD compared to controls (92.8% vs. 98.3%, p<0.001). |
| *Increased movement including restlessness, limb movement, or scratching* | | | | | |
| Fishbein (2018)  Sleep disturbance in children with moderate/severe atopic dermatitis: A case-control study. | Case-control | 19 cases  19 controls | No AD | Actigraphy  Pediatric Sleep Questionnaire | Children with AD had significantly higher frequencies of restless sleep compared to controls (p < 0.01). |
| Reuveni (1999)  Sleep Fragmentation in Children with Atopic Dermatitis. | Case-control | 14 cases  9 controls | No AD | Polysomnography  Scratch electrodes  Self-reported questionnaires filled by caregivers | AD patients experienced bouts of scratching that ranged from 1-19 times per night (1.8 ± 0.6 bouts per hour). Only 15% of overall arousals and awakenings were associated with PSG events such as scratching or other movements, or apnea |
| Benjamin (2004)  The development of an objective method for measuring scratch in children with atopic dermatitis suitable for clinical use. | Case-control | 14 cases  7 controls | No AD | Digital accelerometers  Infrared video recording | Scratching or restlessness was 2-3 times more frequent in children with AD than controls (p < 0.01). |
| Chang (2014)  Atopic Dermatitis, Melatonin, and Sleep Disturbance. | Case-control | 72 cases  32 controls | No AD | Actigraphy  Polysomnography  SCORAD | There was significantly more movement during sleep in patients with AD compared to controls, specifically in the percentage of mobility during sleep time (p<0.05) and limb movement index (p<0.001). |
| Dahl (1995)  Sleep disturbances in children with atopic dermatitis. | Cross-sectional | 59 cases | Normative data | Child Sleep Behavior Scale | No significant differences were found between cases and controls for various types of unusual movement including bruxism, head banging, body rocking, or sleepwalking (p>0.05). |
| *Obstructive sleep apnea & snoring* | | | | | |
| Chng (2004)  Snoring and atopic disease: a strong association. | Cross-sectional | 11,114 cases | Not applicable | Self-reported questionnaire on snoring completed by caregivers | The odds ratio of a child with asthma, allergic rhinitis, and/or AD to having habitual snoring was 7.45 (3.48-15.97), and in particular, the odds ratio of a child with AD to habitual snoring was 1.80 (1.28-2.54). |
| Hu (2018)  Association between obstructive sleep apnea and atopic dermatitis in children: A nationwide, population-based cohort study. | Cross-sectional | 120,736 cases  120,736 controls | No AD | Diagnosis of OSA was provided in Taiwan’s National Health Insurance Research Database | After adjusting for age, sex, urbanization level, and comorbidities, patients with AD had a higher risk of OSA than controls, with an adjusted HR of 1.86 (1.43-2.42). |
| *Nightmares* | | | | | |
| Ramirez (2019)  Association of Atopic Dermatitis with Sleep Quality in Children. | Cohort | 13988 cases | Cases themselves | Standardized questionnaires | 26.2%-49.5% of children with AD experienced regular nightmares. |
| Dahl (1995)  Sleep disturbances in children with atopic dermatitis. | Comparative | 59 cases | Normative data | Child Sleep Behavior Scale | Children with AD did not significantly differ from controls for the frequency of nightmares, sleep terrors, or frightening dreams (p>0.05). |
| *Nocturnal hyperhidrosis* | | | | | |
| Camfferman (2010)  Eczema, sleep, and behavior in children. | Case-control | 77 cases  30 controls | No AD | Sleep Disturbance Scale for Children  Conners Parent Rating Scale-Revised (S)  Child Health Questionnaire  Children’s Dermatology Life Quality Index | A higher percentage of children with AD compared to controls were above the clinical cut-off criteria (T-score >70) for sleep hyperhidrosis (9% [7/77] vs. 7% [2/30]). |
| *Nocturnal enuresis* | | | | | |
| Tsai (2017)  Association between allergic disease, sleep-disordered breathing, and childhood nocturnal enuresis: a population-based case-control study. | Retrospective case-control | 4308 cases with nocturnal enuresis (NE)  4308 controls | No NE | Diagnosis of NE was by inpatient and/or outpatient department visits | Children with atopic dermatitis had a significantly higher odds ratio of 1.23 (1.05-1.43, p=0.008) of having NE compared to controls after adjusting for confounding variables. |
